# Paneth Cell in Adenomas of the Distal Colorectum Is Inversely Associated with Synchronous Advanced Adenoma and Carcinoma

**DOI:** 10.1038/srep26129

**Published:** 2016-05-18

**Authors:** Megan Mahon, Jie Xu, Xianghua Yi, Xiuli Liu, Nan Gao, Lanjing Zhang

**Affiliations:** 1 Department of Pathology, University Medical Center of Princeton, Plainsboro, New Jersey, USA; 2 Department of Pathology, Rutgers Robert Wood Johnson Medical School, New Brunswick, New Jersey, USA; 3 Department of Infectious Diseases, Shanghai Ninth People’s Hospital, Shanghai Jiaotong University, School of Medicine, Shanghai, China; 4 Department of Pathology, Tongji Hospital, Tongji University School of Medicine, Shanghai, China; 5 Department of Anatomic Pathology, Cleveland Clinic, Cleveland, Ohio, USA; 6 Department of Biological Sciences, Rutgers University, Newark, NJ, USA; 7 Department of Chemical Biology, Ernest Mario School of Pharmacy, Piscataway, New Jersey, USA; 8 Cancer Institute of New Jersey, Rutgers University, Piscataway, New Jersey, USA

## Abstract

Recent studies have linked appearance of Paneth cells in colorectal adenomas to adenoma burden and male gender. However, the clinical importance of Paneth cells’ associations with synchronous advanced adenoma (AA) and colorectal carcinoma (CRC) is currently unclear. We performed a comprehensive case-control study using 1,900 colorectal adenomas including 785 from females, and 1,115 from males. We prospectively reviewed and recorded Paneth cell status in the colorectal adenomas consecutively collected between February 2014 and June 2015. Multivariable logistic regression analyses revealed that, in contrast to the adenomas without Paneth cells, the Paneth cell-containing adenomas at distal colorectum were inversely associated with presence of a synchronous AA or CRC (odds ratio [OR] 0.39, *P* = 0.046), whereas no statistical significance was reached for Paneth cell-containing proximal colorectal adenomas (*P* = 0.33). Synchronous AA and CRC were significantly associated with older age (60 + versus <60 years, OR 1.60, *P* = 0.002), male gender (OR 1.42, *P* = 0.021), and a history of AA or CRC (OR 2.31, *P* < 0.001). However, synchronous CRC was not associated with Paneth cell status, or a history of AA or CRC. Paneth cell presence in the adenomas of distal colorectum may be a negative indicator for synchronous AA and CRC, and seems to warrant further studies.

Colorectal cancer (CRC) is the third leading cause of cancer-related deaths among both men and women in the United States^1^. Colonoscopy guidelines recommend that individuals should start having a colonoscopy at the age of 50 years, and a potential follow-up colonoscopy depending on the endoscopic findings, particularly the polyp number and characteristics^2^,^3^. Certain adenoma characteristics have been associated with an increased CRC risk, including polyp size greater than or equal to 1 cm, villous histology and high-grade dysplasia^2^,^3^,^4^,^5^,^6^,^7^,^8^. An adenoma with one or more of the 3 characteristics is considered as advanced adenoma (AA, also known as advanced neoplasia) and should be followed up within 3 years, according to the recent update of the U.S. Multi-society Task Force on Colorectal Cancer^2^. Identification of markers for AA may help with the prevention, early identification and treatment of CRC.

Paneth cells are normally present in the small intestine, proximal colon and transverse colon, and contribute to mucosal innate immunity by exerting a number of anti-microbial effects^9^,^10^,^11^. Genetic studies have indicated that Paneth cells upregulate the production of lysozymes, phospholipase A2, the Apc/beta-catenin/Tcf pathway, WNT, and CD166, during colonic tumorigenesis^11^,^12^,^13^,^14^,^15^,^16^,^17^. Paneth cells are also critical for intestinal stem cell homeostasis, as shown by our and others’ works^18^,^19^,^20^. Recently, more attentions have been focused on the role of Paneth cells in CRC development and diagnosis^9^.

The detection of Paneth cells in colorectal adenomas was reported as early as 1967^10^. The reported frequencies of Paneth cell presence in colorectal adenomas vary significantly, ranging from 0.2 to 39% 15,21,22,23,24. The current consensus view is that Paneth cells are exclusively seen in normal proximal colon (right and transverse colon), and in the injured distal colon and rectum such as the one in inflammatory bowel disease^7^,^21^,^25^,^26^,^27^. In terms of the association of Paneth cells with CRC, recent studies reported somewhat contradicting data. One study identifies Paneth cells in the junctional mucosa of 45% of CRC^28^, while in another study Paneth cell presence was seen in only 2.5% of CRC and 38.5% of conventional adenomas (tubular, villous, or tubulovillous adenomas)^15^. Paneth cells were also found more frequently seen in CRCs than in tubular adenomas^26^. Finally, an association was recently reported between Paneth cell containing adenomas and male gender as well as the adenoma burden^21^. However, it is still not clear whether presence of Paneth cells in colorectal adenomas is associated with presence of synchronous AA or synchronous CRC. This case-control study was specifically designed to address these questions.

## Results

**Figure 1:**
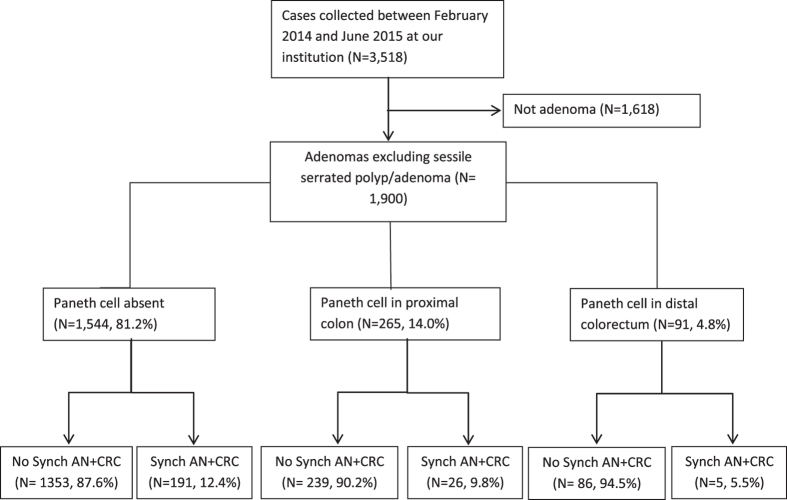
Flow chart of the case distribution. Synch AA + CRC: synchronous advanced neoplasia and colorectal cancer.

**Figure 2:**
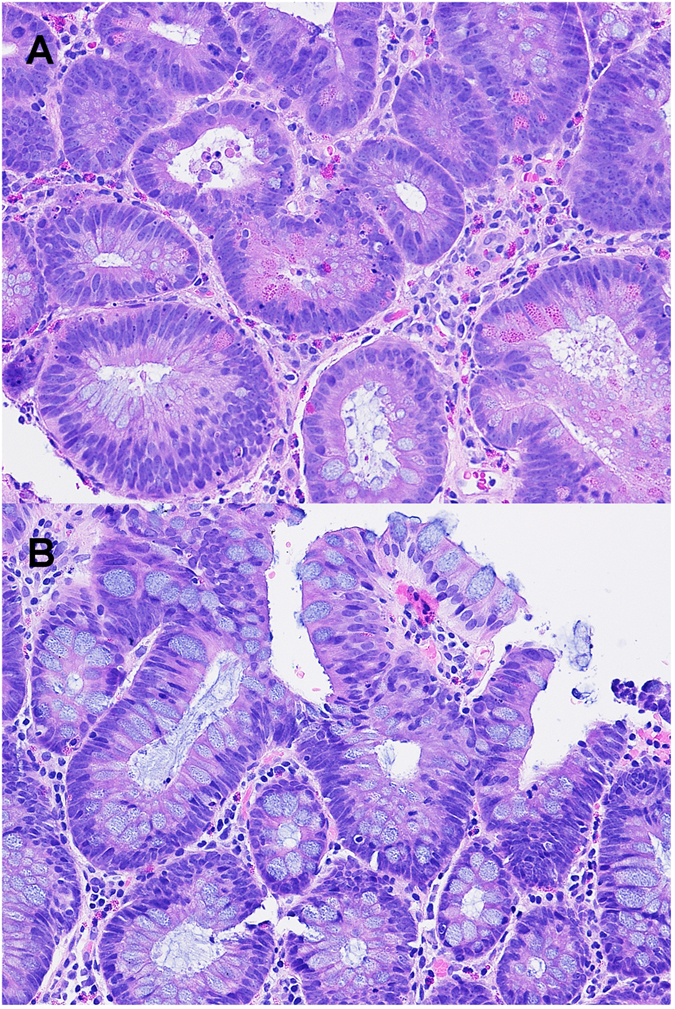
(**A**) A tubular adenoma showing many Paneth cells with irregular distribution and coarse eosinophilic granules facing the lumen (200x). The adenomatous cells also show frequent mitoses (left upper corner), unclear hyperchromatia, loss of polarity, crowding/overlapping and pencil-like or round nuclei. (**B**) A tubular adenoma showing rare eosinophils and no Paneth cells (200x).

As shown in Fig. 1, a total of 3518 cases collected between February 2014 and June 2015 were identified in our study, with 1900 qualified conventional (non-AA) adenomas and AA including 785 from females (17.2% with Paneth cells), and 1115 from males (19.8% with Paneth cells). Compared to the fine, evenly distributed granules and bilobed or trilobed nuclei of eosinophils, Paneth cells show coarse, lumen-facing granules and single round nuclei (Fig. 2A). Overall, 18.7% of the colorectal adenomas showed evidence of Paneth cells, with Fig. 2B as an example.

Demographic and clinical characteristics pertaining to the presence of Paneth cells in the colorectal adenomas were obtained and reviewed (Table 1). Seventeen hundred and sixty five patients (92.89%) were 50 years of age or older. A history of AA or CRC was significantly associated with Paneth cell absence (*P* = 0.009) and location of Paneth-cell-containing adenomas (*P* = 0.029). Table 1 also shows that the adenoma location had a significant association with Paneth cell status (Presence versus absence, *P* < 0.001).

As Table 2 shows, presence of a synchronous AA or CRC was associated with ages 60 years and older (OR: 1.78, *P* < 0.001), 65 years and older (OR: 1.58, *P* < 0.001), male sex (OR: 1.3, *P* = 0.032) and history of AA or CRC (OR: 2.83, *P* < 0.001), but not location of adenoma (OR: 0.89, *P* = 0.072). Paneth cell presence (OR: 0.68, *P* = 0.054) and Paneth-cell-containing adenoma in the distal colorectum (OR: 0.41, *P* = 0.057) tended to link to a synchronous AA or CRC. Our multivariable logistic regression analysis (LRA) revealed that Paneth-cell-containing adenoma of the distal colorectum (OR: 0.39, *P* = 0.046) was inversely associated with a synchronous AA or CRC, in addition to older age (60 +  years), male sex and a history of AA or CRC (Table 2). A separate multivariable LRA showed that lack of Paneth cells in adenomas had a trend to link to presence of synchronous AA or CRC (*P* = 0.054, Table 2).

We then explored the factors potentially associated with synchronous CRC (Table 3). Only history of AA or CRC was found associated with synchronous CRC (OR: 3.47, *P* = 0.033). Compared with adenomas without Paneth cells, Paneth cell-containing adenomas in the proximal colon (*P* = 0.157) and in the distal colorectum (*P* = 0.797) were not associated with synchronous CRC.

## Discussion

This case-control study is one of the first studies to investigate the association between Paneth cell presence in colorectal adenomas and synchronous AA and CRC. Our multivariable modeling on the population of conventional adenomas and AA suggested that, compared to the adenomas without Paneth cells, Paneth-cell-containing adenomas at the distal colorectum were inversely (61% likelihood reduction) associated with a synchronous AA or CRC, but not associated with synchronous CRC.

AA is associated with a higher risk of developing CRC and hence warrants a shorter follow-up interval^2^. Therefore, identification of the factors associated with synchronous AA and CRC may help screen for the patients with a higher likelihood of having an AA and/or CRC. However, despite the important role of Paneth cells in intestinal stem cell homeostasis^18^,^19^,^20^, few studies have investigated the association between synchronous AA/CRC and Paneth cell presence status in colorectal adenomas. Our data seem interesting because they show an inverse relationship between Paneth-cell-containing adenoma at the distal colorectum and presence of a synchronous AA or CRC. The findings are contradictory to the prevailing theories that Paneth cells contribute to the development of colonic epithelial neoplasia through various cellular and molecular mechanisms^19^,^22^,^29^,^30^. This inverse association provides new and perhaps also important information to the field, and raises the question regarding how, if at all, Paneth cells reversely link to the synchronous AA and CRC development in the distal colorectum. Consistent with the findings of this study, our preliminary data of a separate study show a lower frequency of Paneth cell presence in adenomas with villous histology and/or high-grade dysplasia than in conventional adenomas (unpublished data, Xu and Zhang). It is also noteworthy that one of the earlier studies did not reveal any association between Paneth cell presence and the histologic features of AA^21^.

We did not find any association between race and Paneth cells. This is in contrast to the earlies study showing that Paneth cells are more commonly seen in Japanese descendants and White residents of Hawaii compared to native Japanese^23^. This discrepancy may be due to our patient population that consisted of 78.4% Whites and 11% Asians. Race also did not have any association with synchronous AA or CRC, despite the earlier finding that African Americans have an increased risk of CRC^31^. This discrepancy may be attributed to the fact that only few CRC cases were included in our cohort, along with the predominance of White patients in our study population. The sample size may be too small to reveal a potential association.

Some of this study’s strengths are noteworthy. First, our work appears to fill in the knowledge gap on the association between Paneth cell presence in adenomas and presence of a synchronous AA or CRC. The identified inverse association suggests that Paneth cells in the distal colorectum may be a negative indicator for synchronous AA and CRC. More follow-up studies are needed to confirm our findings. Second, the large-scale of this study seemed to have provided sufficient statistical power in some aspects, and may explain the unique factor associated with presence of a synchronous AA or CRC. Indeed, our study also confirms the reported 0.2 to 39% prevalence of Paneth cell presence in colorectal adenomas^15^,^21^,^22^,^23^,^24^, and supports the prior findings that Paneth cells were more commonly seen in the proximal colon^7^,^21^,^25^,^26^,^27^. Third, case-control studies like ours would be able to dissect the association between the factors and outcomes^2^,^3^,^32^, and may provide a higher level of evidence than case-series studies.

This study had several potential limitations. First, the case-control studies could only examine potential associations, not causality. Therefore, a cohort study is needed to examine whether the presence of Paneth cell in distal colorectum would decrease the risk of synchronous AA and CRC. Second, we used AA as a term combining three characteristics. Separating AA into three distinct categories of high grade dysplasia, size greater than or equal to 1 cm and villous histology could affect the results. Third, none of the potential factors was found associated with presence of a synchronous CRC including some well-known cancer risk factors such as age and adenoma location. One explanation is the small number of the synchronous CRC cases included in our study. In fact, only 16 of our 1900 adenomas had a synchronous CRC (<1%), and none of these cases had presence of Paneth cells. A study with more synchronous CRC cases is needed to validate our findings. Last, we used the data from only one institution and a selection bias may have been resulted in. As discussed earlier, some of the differences between our study and the earlier one^21^ may be explained by the study population differences. Taken together, caution should be taken while generalizing the findings of this study.

In conclusion, Paneth cell presence in the adenomas at the distal colorectum is inversely associated with presence of a synchronous AA or CRC, and may be used as a negative indicator for synchronous AA and CRC. Our findings also suggest an alternative hypothesis that Paneth cells in adenomas of the distal colorectum may link to the suppression of adenoma progression to AA and/or CRC. Future studies are needed to confirm and explain our findings.

## Materials and Methods

The study protocol was reviewed and approved by the Institutional Review Board (IRB) of University Medical Center of Princeton at Plainsboro, Plainsboro, New Jersey, USA. The study adheres to the STrengthening the Reporting of OBservational studies in Epidemiology (STROBE) statement and was carried out in accordance with the approved IRB protocol, and relevant guidelines and regulations. Informed consent was obtained from all subjects. Consecutive colorectal polyp cases collected at the University Medical Center of Princeton at Plainsboro, New Jersey between February 2014 and June 2015 were reviewed by a pathologist (LZ) and one of his departmental colleagues, and prospectively included in the Princeton Colorectal Polyp Cohort (PCPC) which was started in August 2012. The patients who had an inflammatory bowel disease were excluded from the PCPC. The patient demographics and clinical characteristics obtained for each case consisted of age, sex, self-reported race (White, Hispanic, African American, Asian and others), location of the polyp in the colon, Paneth cell status (started in February 2014), CRC history and polyp history, and synchronous colorectal lesions. The inclusion criteria for this case-control study were colorectal adenomas with a known status of Paneth cell presence. Due to the uncertain biological behaviors of sessile serrated polyp/adenoma, it was not included in this study. Both conventional (non-advanced) and AA were included in the study. As prescribed in the recent update of the US multi-society task force on colorectal cancer, AA was defined as adenoma with high-grade dysplasia, greater than or equal to 1 cm in size, or with villous histology^2^. The term of proximal colon included right colon and transverse colon, while distal colorectum included descending colon, sigmoid colon and rectum. Therefore, the cutoff point between the proximal colon and distal colonrectum was splenic flexure. The positive history of CRC was defined as a history of CRC given by the caring gastroenterologist or a diagnosis of CRC rendered at our institution three months or more prior to the adenoma-diagnosis time. We used the term synchronous to describe any additional polyps identified during the same endoscopic procedure.

The tissue was processed using standard histological protocols and stained using hematoxylin and eosin. At least 3 levels for each biopsy were examined according to the routine pathology examination protocol in the USA. Presence of one or more Paneth cells is considered as positive for Paneth cells. STATA IC version 11 (Stata Corp, College Station, TX, USA) was used for the statistical analyses as described before^33^. Exact LRA was performed for the variables with no cases (0) in a computation cell/subgroup. If a variable met the criterion of having a *P-*value of less than or equal to 0.1 as determined by the univariate LRA, it would be included in the multivariable LRA. In the univariate and multivariable analyses, the control group included adenoma cases that did not have synchronous AA or synchronous CRC, while the case group included adenomas with at least one synchronous AA or CRC (Table 2), or with at least one synchronous CRC (Table 3).

## Additional Information

**How to cite this article**: Mahon, M. *et al.* Paneth Cell in Adenomas of the Distal Colorectum Is Inversely Associated with Synchronous Advanced Adenoma and Carcinoma. *Sci. Rep.* **6**, 26129; doi: 10.1038/srep26129 (2016).

## Figures and Tables

**Table 1:** Demographic and clinical characteristics of the included colorectal adenomas.

General Characteristics			Paneth cell status		Paneth cell status-3 categories				
		sum	Absence	Presence	*P*-value	Absence	Present in proximal colon	Present in distal colorectum	*P*-value
Age, year									
	<39	20	18 (90)	2 (10)	0.005*	18 (90)	2 (10)	0	0.027*
	40–49	115	98 (85.22)	17 (14.78)		98 (85.22)	13 (11.3)	4 (3.48)	
	50–59	681	572 (83.99)	109 (16.01)		572 (83.99)	86 (12.63)	23 (3.38)	
	60–69	629	512 (81.40)	117 (18.60)		512 (81.40)	84 (13.35)	33 (5.25)	
	>70	455	344 (75.60)	111 (24.40)		344 (75.60)	80 (17.58)	31 (6.81)	
Gender									
	Female	785	650 (82.8)	135 (17.2)	0.149	650 (82.8)	96 (12.23)	39 (4.97)	0.191
	Male	1,115	894 (80.18)	221 (19.82)		894 (80.18)	169 (15.16)	52 (4.66)	
History of CRC	Positive	37	32 (86.49)	5 (13.51)	0.526*	32 (86.49)	1 (2.7)	4 (10.81)	0.027*
	Negative	1863	1512 (81.16)	351 (18.84)		1512 (81.16)	264 (14.17)	87 (4.67)	
History of AA or CRC	Positive	169	150 (88.76)	19 (11.24)	**0.009**	150 (88.76)	13 (7.69)	6 (3.55)	**0.029**
	Negative	1731	1394 (80.53)	337 (19.47)	1394 (80.53)	252 (14.56)	85 (4.91)		
Synchronous CRC	Positive	16	16 (100)	0	**** 0.054*	16 (100)	0	0	**** 0.222*
	Negative	1884	1528 (81.1)	356 (18.9)	1528 (81.1)	265 (14.07)	91 (4.83)		
Presence of a Synchronous AA or CRC	Positive	222	191 (86.04)	31 (13.96)	0.052	191 (86.04)	26 (11.71)	5 (2.25)	0.082*
	Negative	1678	1353 (80.63)	325 (19.37)		1353 (80.63)	239 (14.24)	86 (5.13)	
Race	White	1489	1208 (81.13)	281 (18.87)	0.894	1208 (81.13)	218 (14.64)	63 (4.23)	0.151*
	Hispanic	16	14 (87.5)	2 (12.5)		14 (87.5)	2 (12.5)	0	
	African American	40	34 (85)	6 (15)		34 (85)	2 (5)	4 (10)	
	Asian	209	167 (79.9)	42 (20.1)		167 (79.9)	26 (12.44)	16 (7.66)	
Location	Right colon	760	567 (74.61)	193 (25.39)	**<0.001**	567 (74.61)	193 (25.39)	0	NA
	Transverse colon	346	279 (80.64)	67 (19.36)		279 (80.64)	67 (19.36)	0	
	Distal colorectum	772	681 (88.21)	91 (11.79)		681 (88.21)	0	91 (11.79)	
Sum		1900	1544 (81.26%)	356 (18.74)		1544 (81.26%)	265 (13.95)	91 (4.79)	

Note: All *P*-values were calculated using Chi-square test; CRC: Colorectal carcinoma; AA: advanced adenoma; (*) Fisher’s exact test used.

**Table 2:** Factors associated with the presence of synchronous advanced adenomas or carcinoma in patients with colorectal adenoma.

Factor		Univariate		Multivariable model 1 (N = 1878)		Multivariable model 2 (N = 1878)	
		OR (95% CI)	*P*-value	OR (95% CI)	*P*-value	OR (95% CI)	*P*-value
Age, year	60 + vs<60	1.78 (1.39–2.28)	**<0.001**	1.60 (1.18–2.17)	**0.002**	1.59 (1.17–2.15)	**0.003**
	65 + vs <65	1.58 (1.25–2.01)	**<0.001**				
Gender	Male vs Female	1.3 (1.02–1.66)	**0.032**	1.42 (1.05–1.92)	**0.021**	1.43 (1.06–1.93)	**0.019**
History of CRC	Yes vs None	1.09 (0.43–2.76)	0.85				
History of AA or CRC	Yes vs None	2.83 (2.02–3.98)	**<0.001**	2.31 (1.55–3.45)	**<0.001**	2.30 (1.54–3.44)	**<0.001**
Race		0.92 (0.80–1.04)	0.184				
Location of Adenoma	Distal vs Proximal colon	0.89 (0.78–1.01)	0.072	1.05 (0.89–1.25)	0.55	1.01 (0.86–1.19)	0.088
PC Status	Present vs Absent	0.68 (0.45–1.00)	0.054			0.67 (0.44–1.01)	0.054
PC Status–3 Categories	PC in proximal colon vs No PC	0.77 (0.5–1.19)	0.237	0.79 (0.50–1.26)	0.33		
	PC in distal colorectum vs no PC	0.41 (0.17–1.03)	0.057	0.39 (0.15–0.98)	**0.046**		

Note: AA: advanced adenoma; OR: Odds ratio; CI: confidence intervals; CRC: Colorectal carcinoma; PC: Paneth Cells; vs: versus.

**Table 3:** Potential factors associated with the presence of synchronous carcinoma in patients with colorectal adenoma.

Factor		Univariate		Multivariable (N = 1878)	
		OR (95% CI)	*P*-value	OR (95% CI)	*P*-value
Age, yr	60 + vs<60	1.66 (0.58–4.80)	0.35		
	65 + vs <65	0.89 (0.32–2.45)	0.82		
Gender	Male vs Female	0.54 (0.20–1.47)	0.23		
History of CRC*	Yes vs None	2.23 (0–13.52)	1		
History of AA or CRC	Yes vs None	3.47 (1.11–10.89)	**0.033**	3.15 (0.73–10.57)	0.123
Race		1.30 (0.88–1.92)	0.19		
Location of Adenoma	Distal vs Proximal colon	1.27 (0.72–2.27)	0.41		
PC Status*	Present vs Absent	0.19 (0–1.12)	0.071	0.21 (0–1.22)	0.091
PC Status-3 Categories*	PC in proximal colon vs No PC	0.26 (0–1.51)	0.157		
	PC in distal colorectum vs No PC	0.75 (0–4.44)	0.797		

Note: OR: Odds ratio; CI: confidence intervals; AA: advanced adenoma; CRC: Colorectal carcinoma; vs: versus; (*) Exact logistic regression analysis used.
